# Association between urea-to-creatinine ratio trajectories and clinical outcomes in chronic critical illness patients: a retrospective cohort study

**DOI:** 10.3389/fnut.2026.1776277

**Published:** 2026-07-06

**Authors:** Jia Song, Lu Shen, Jiayi Li, Mingkun Yang, Lina Chen, Yikuan Shen, Qiancheng Luo, Shijin Gong, Weihang Hu, Minjia Wang

**Affiliations:** 1 Department of Critical Care Medicine, Zhejiang Hospital, Hangzhou, Zhejiang, China; 2 The Second School of Clinical Medicine, Zhejiang Chinese Medical University, Hangzhou, Zhejiang, China; 3 Department of Critical Care Medicine, Pudong Gongli Hospital, Shanghai University of Medicine and Health Sciences, Shanghai, China

**Keywords:** chronic critical illness, group-based trajectory model, MIMIC-IV database, prognosis, urea-to-creatinine ratio

## Abstract

**Background:**

Chronic critical illness (CCI) is characterized by persistent organ dysfunction and profound catabolism, yet reliable biomarkers to monitor catabolic burden are lacking. The urea-to-creatinine ratio (UCR) has been proposed as a biochemical signature of critical illness-associated catabolism. This study aimed to identify longitudinal UCR trajectory patterns in CCI patients and to evaluate their association with mortality.

**Methods:**

We conducted a retrospective cohort study using the MIMIC-IV database. Adult patients (≥18 years) who met the criteria of CCI in their first ICU admission period were included. CCI was defined as an ICU length of stay over 14 days and coexists with persistent organ dysfunction at day 14. Patients with serum creatinine > 4 mg/dL at ICU admission, renal replacement therapy, gastrointestinal bleeding, or obstetric admissions were excluded. Daily UCR from ICU days 1–14 was modeled using group-based trajectory modeling (GBTM). Outcomes were 28-day, 90-day, and 365-day mortality after CCI diagnosis. Kaplan–Meier survival curves and Cox proportional hazards models were applied to analyze the association between UCR trajectories and outcomes. Subgroup analyses were conducted to further validate the robustness of the results.

**Results:**

A total of 1718 CCI patients were included. Four distinct UCR trajectory groups were identified: trajectory 1 (low-level, stable), trajectory 2 (low-level, slow increase), trajectory 3 (mid-level, slow increase), and trajectory 4 (high-level, rapid increase). Kaplan–Meier analysis showed that mortality increased progressively from trajectory 1 to trajectory 4. In a fully adjusted Cox proportional hazards model, the 28-day, 90-day, and 365-day mortality risks were significantly higher in trajectory 2–4 groups compared with trajectory 1 group. Subgroup analyses revealed a statistically significant interaction between age and UCR trajectory groups.

**Conclusion:**

Longitudinal UCR trajectories provide important prognostic information in CCI patients and are independently associated with short- and long-term mortality. Monitoring UCR trends during the early ICU course may facilitate metabolic phenotyping and risk stratification of CCI patients.

## Introduction

Over the past decades, advances in intensive care medicine have dramatically improved survival rates of critically ill patients. Many patients are now discharged alive from intensive care units (ICUs). However, a subset of these survivors does not recover quickly and instead develops chronic critical illness (CCI), a state of persistent organ dysfunction requiring long-term life support (1). Studies show that CCI occurs in approximately 33%–40% of ICU survivors (2–4), with incidence rates approaching 50% among those recovering from sepsis (5). Patients with CCI typically experience longer ICU and hospital lengths of stay (LOS), higher rates of discharge to long-term health care facilities, and increased mortality (6–9).

One proposed pathophysiological mechanism underlying CCI is persistent inflammation, immunosuppression, and catabolism syndrome (PICS), which has been described as an endotype of CCI (10–12). Notably, an estimated 30%–50% of CCI patients show evidence of PICS (13). PICS is characterized by a self-perpetuating cycle of organ failure, inflammation, and immunosuppression, leading to recurrent infections, metabolic derangements, and muscle wasting (14). Catabolism is a central feature of PICS: ongoing catabolic activity ultimately results in severe muscle wasting and associated weakness, which in turn impairs patient outcomes (15, 16). Identifying reliable markers of catabolism would greatly aid in evaluating the efficacy of future interventions. However, monitoring catabolism remains challenging because no biomarker currently offers acceptable sensitivity or specificity (17). Therefore, there is a critical need for a reliable, standardized, and readily available indicator to monitor the overall catabolic burden and metabolic trajectory in CCI patients.

The urea-to-creatinine ratio (UCR) is a long-established measure of catabolism, which has recently seen renewed interest (18). A raised UCR has been associated with persistent critical illness and critical illness-associated catabolism as it reflects both nitrogen liberation and reduced muscle mass (17, 19, 20). In a trauma-ICU cohort, Haines et al. identified UCR as a metabolic signature for the ongoing catabolic state characterizing persistent critical illness (19). Moreover, several studies have supported the use of UCR as a marker of muscle wasting (21–23). However, during ICU stays, UCR undergoes dynamic changes over time, which may reflect changes in the catabolic state at different stages of critical illness. Research on longitudinal UCR trajectories in CCI patients remains limited. Group-based trajectory modeling (GBTM) offers a framework to capture patient heterogeneity and to explore such dynamic changes over time (24). By applying GBTM to UCR data in CCI patients, distinct trajectory subgroups can be identified; these subtypes may carry significant prognostic implications and could inform targeted interventions.

Therefore, the present study aimed to identify longitudinal UCR trajectory patterns among CCI patients and to explore their associations with clinical outcomes. Through this approach, we sought to evaluate the clinical utility of UCR trajectories as a simple, readily available biomarker reflecting metabolic stress and overall physiological condition in this population. Ultimately, this evidence may inform future research on personalized risk stratification strategies aimed at improving patient outcomes.

## Materials and methods

### Data source

Data for this study were sourced from the MIMIC-IV (version 3.1) database, a publicly available repository of de-identified health records from ICU admissions at Beth Israel Deaconess Medical Center (BIDMC) between 2008 and 2022 (25). To obtain data access, we completed the National Institutes of Health (NIH) human-subjects training and passed the Collaborative Institutional Training Initiative Program exam. The author Jiayi Li has completed the Collaborative Institutional Training Program exam (certification number: 69660239) and the research resource has received institutional review board (IRB) approval at the BIDMC. All patient data were fully de-identified, so informed consent is not required. This study followed the Strengthening the Reporting of Observational Studies in Epidemiology (STROBE) guidelines for observational research (26).

### Study population

This study included adult patients (aged ≥ 18 years) with CCI who were admitted to the ICU for the first time. CCI was defined as a long-term critical illness with organ dysfunction during the ICU stay, and we used the diagnostic criteria proposed by Gardner et al. (2): (1) ICU duration ≥ 14 days; (2) evidence of persistent organ dysfunction [defined as a cardiovascular Sequential Organ Failure Assessment (SOFA) score ≥ 1 or a SOFA score ≥ 2 in any other organ system] at day 14. The exclusion criteria were: (1) serum creatinine > 4 mg/dL at ICU admission; (2) received renal replacement therapy (RRT) within 14 days after ICU admission; (3) occurrence of gastrointestinal bleeding at ICU admission; (4) ICU admission for obstetric reasons (volume expansion during pregnancy can lower urea levels). Finally, a total of 1718 patients were retained for analysis.

### Data extraction

Data extraction was performed using Navicat Premium 17.0 with structured query language (SQL) to query the MIMIC-IV database. Laboratory values (except for urea and creatinine) were recorded using the first measurement obtained after ICU admission, whereas vital signs were summarized as the mean values from the first 24 h of the ICU stay. We collected a broad range of patient characteristics, including: demographic information [age, race, gender, and body mass index (BMI)]; vital signs [heart rate (HR), systolic blood pressure (SBP), diastolic blood pressure (DBP), mean blood pressure (MBP), and respiratory rate (RR)]; disease severity scores [Glasgow Coma Scale (GCS), Acute Physiology Score III (APS III), baseline SOFA (highest SOFA within first 24 h of ICU admission), Charlson Comorbidity Index, and Oxford Acute Severity of Illness Score (OASIS)]; comorbidities [hypertension, diabetes, congestive heart failure, liver disease, chronic pulmonary disease, chronic kidney disease (CKD), cerebrovascular disease, acute kidney injury (AKI) and sepsis]; laboratory data [anion gap, bicarbonate, chloride, sodium, potassium, calcium, magnesium, blood urea nitrogen (BUN), creatinine, platelet, hemoglobin, hematocrit, red cell distribution width (RDW), white blood cell (WBC), glucose, aspartate aminotransferase (AST), alanine aminotransferase (ALT), direct bilirubin (DBIL), activated partial thromboplastin time (APTT), prothrombin time (PT), international normalized ratio (INR), partial pressure of oxygen (PaO_2_), partial pressure of carbon dioxide (PaCO_2_), ratio of partial pressure of oxygen to fraction of inspired oxygen (P/F ratio) and lactate]; treatment (use of invasive mechanical ventilation, norepinephrine, and epinephrine); outcomes (hospital LOS, ICU LOS, hospital mortality, ICU mortality, 28-day mortality, 90-day mortality, and 365-day mortality). CKD was identified using ICD-9 and ICD-10 (code 585 and N18) in the MIMIC-IV database.

Variables with more than 20% missing values were excluded from analysis. For the remaining variables, missing values were handled using multiple imputation by chained equations (MICE) in R. Ten imputed datasets were generated, applying imputation methods appropriate to each variable type. Odds ratios and variance–covariance matrices were estimated separately for each imputed dataset and then pooled using Rubin’s rules to obtain final combined estimates. Finally, results were consolidated across the 10 imputations according to Rubin’s rules (27). Details of the missing data are shown in Supplementary Table 1.

For each day from 1 through 14 of the ICU stay, we calculated the UCR using the first available BUN and creatinine values recorded (both in mg/dL).

### Outcome indicators

In this study, the endpoints consisted of 28-day, 90-day and 365-day mortality after patients were diagnosed with CCI (at day 14 in ICU).

### Group-based trajectory modeling

In the study, GBTM was employed to examine the variations in UCR from day 1 to day 14 after ICU admission among participants. GBTM is a semiparametric model tailored for analyzing longitudinal data and operates under the assumption that the population is comprised of discrete, identifiable subgroups. This methodology facilitates the identification of distinct categories within the population, each characterized by homogeneous UCR trajectory profiles. GBTM categorized UCR trajectories into one to five subgroups to best represent the changes over the specified period. Determination of the ideal number of trajectory groupings was informed by a combination of criteria, including: (1) Bayesian Information Criterion (BIC) and Akaike Information Criterion (AIC), with lower values indicating better fit; (2) Average posterior probability (AvePP), >0.7 suggest reliable subgroup classification; (3) Proportion of patients in each trajectory group > 5%; (4) Odds of correct classification (OCC), the minimum OCC value should be greater than 5.0, with higher values indicating better classification; (5) A comprehensive evaluation of model simplicity and clinical interpretability.

### Statistical analysis

In baseline description, normality testing of continuous variables was performed using the Shapiro–Wilk test. Normally distributed data were presented as mean ± standard deviation (SD), and between-group comparisons were performed using one-way ANOVA. Non-normally distributed data were expressed as median (interquartile range), and comparisons between groups were conducted using the Kruskal–Wallis test. Categorical variables were presented as frequencies and percentages, and comparisons were made using the Chi-squared (χ^2^) test or Fisher’s exact test. To address missing data in our variables, we employed multiple imputation techniques, thereby helping to ensure the integrity of our analyses. Survival analysis was conducted using the Kaplan–Meier curves with the log-rank test based on UCR trajectories at 28-day, 90-day and 365-day mortality after CCI diagnosis.

As with all observational studies, estimation of the effect of an exposure on an outcome needs to consider potential confounding. Directed acyclic graphs (DAGs) is a useful tool for visually representing the complex interplay of clinical factors affecting exposure and outcome relationships (28). We used DAGs to display the confounding variables that may affect both the exposure (UCR trajectory groups) and the outcome (mortality). These confounding variables were identified from among the covariates in our study (e.g., age, gender, BMI, albumin infusion, comorbidities, vital signs, disease severity scores, clinical test results, etc.). Furthermore, we conducted Cox proportional hazards models to examine the relationship between UCR trajectory groups and 28-day, 90-day and 365-day mortality, with results reported as hazard ratios (HRs) and 95% confidence intervals (CIs). Two models were conducted, one was an unadjusted crude model, and the second was a fully adjusted model (adjusted for confounding variables identified by the DAGs).

Ultimately, we performed multiple subgroup analyses to explore potential influencing factors of the relationship between UCR trajectory groups and 28-day, 90-day and 365-day mortality. These analyses stratified patients by sex (male vs. female), age (≤65 years vs. >65 years), BMI (<20 vs. 20–25 vs. >25), SOFA score (<6 vs. ≥6), and the presence of comorbidities (yes vs. no). We applied a Bonferroni correction to adjust for multiple comparisons, and we conducted interaction testing in the subgroup analyses. We also conducted additional exploratory analyses, an expanded sensitivity cohort including patients with admission creatinine > 4 mg/dL and/or RRT was explored to assess the robustness of the UCR-mortality association in severe renal dysfunction. All statistical analyses were performed using R (version 4.4.3)^1^. A two-sided *p*-value < 0.05 was considered statistically significant.

## Results

### UCR trajectories and baseline characteristics

**FIGURE 1:**
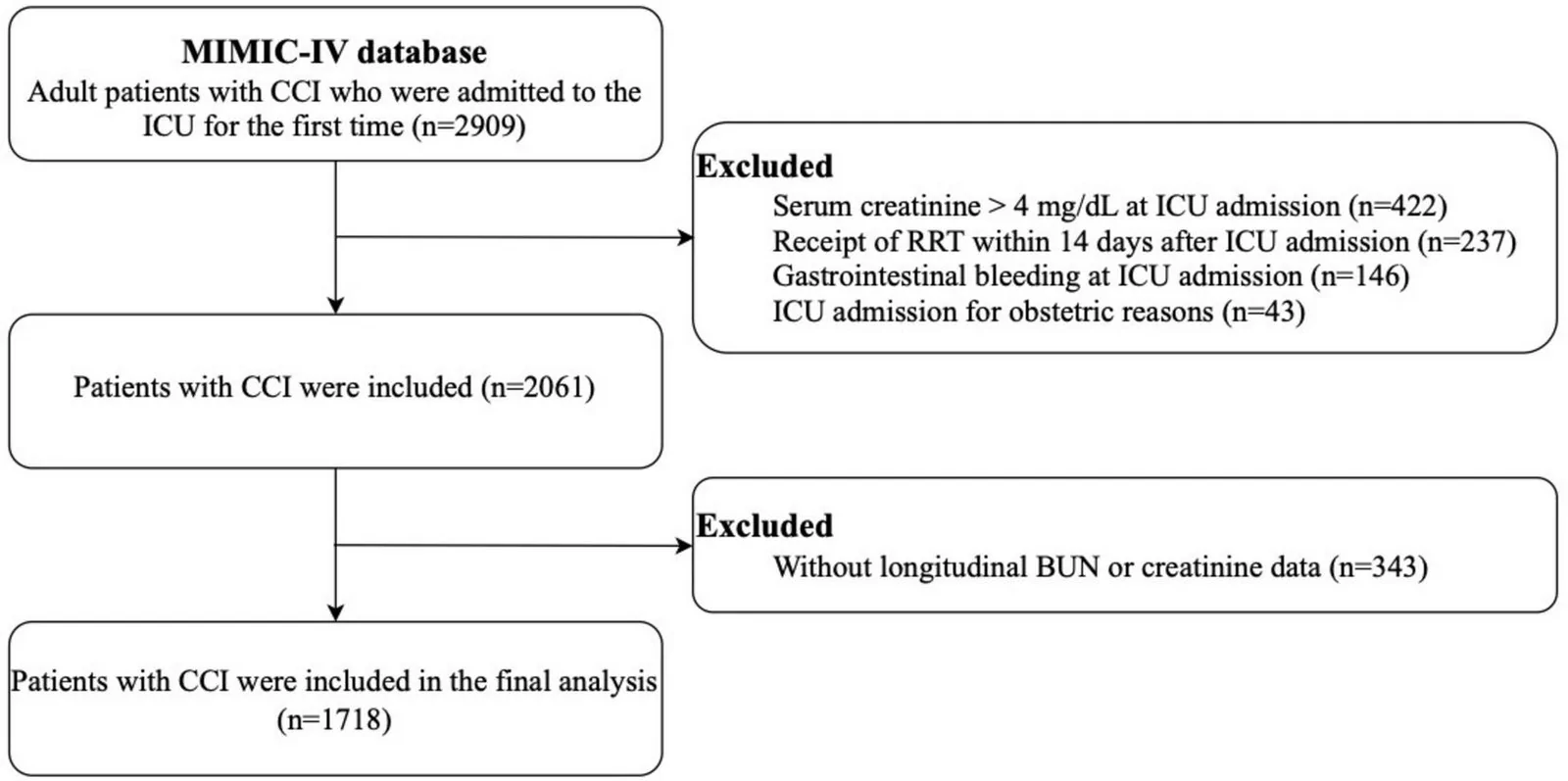
Flowchart of patient selection process.

In this study, a total of 1718 CCI patients were included from the MIMIC-IV database. Figure 1 shows the flowchart of patient selection for this study. Table 1 presents the model fit statistics and AvePP used to determine the optimal number of UCR trajectory groups. The 4-group and 5-group trajectory models had similar AIC/BIC values and were among the best-fitting candidate models. Furthermore, the proportion of the population in each group exceeded 5%, and both AvePP and the minimum OCC (correct classification rate) met the required thresholds. After considering the overall interpretability and simplicity of the model, we selected the 4-group trajectory model.

**TABLE 1:** Performance of the group-based trajectory model for UCR trajectories.

Trajectories	BIC	AIC	AvePP	Minimum OCC	Class proportion
1	230865.37	230832.42	1.00	NaN	100.0%
2	183749.39	183700.35	0.98/0.98	35.30	63.2%/36.8%
3	177599.60	177523.31	0.98/0.97/0.97	40.61	35.4%/45.3%/19.3%
4	174595.21	174491.68	0.97/0.95/0.96/0.97	34.32	22.8%/37.3%/29.5%/10.4%
5	173152.94	173022.16	0.96/0.94/0.93/0.97/0.96	39.33	13.5%/27.4%/23.2%/8.9%/27.1%

BIC, Bayesian Information Criterion; AIC, Akaike Information Criterion; AvePP, average posterior probability; OCC, odds of correct classification.

**FIGURE 2:**
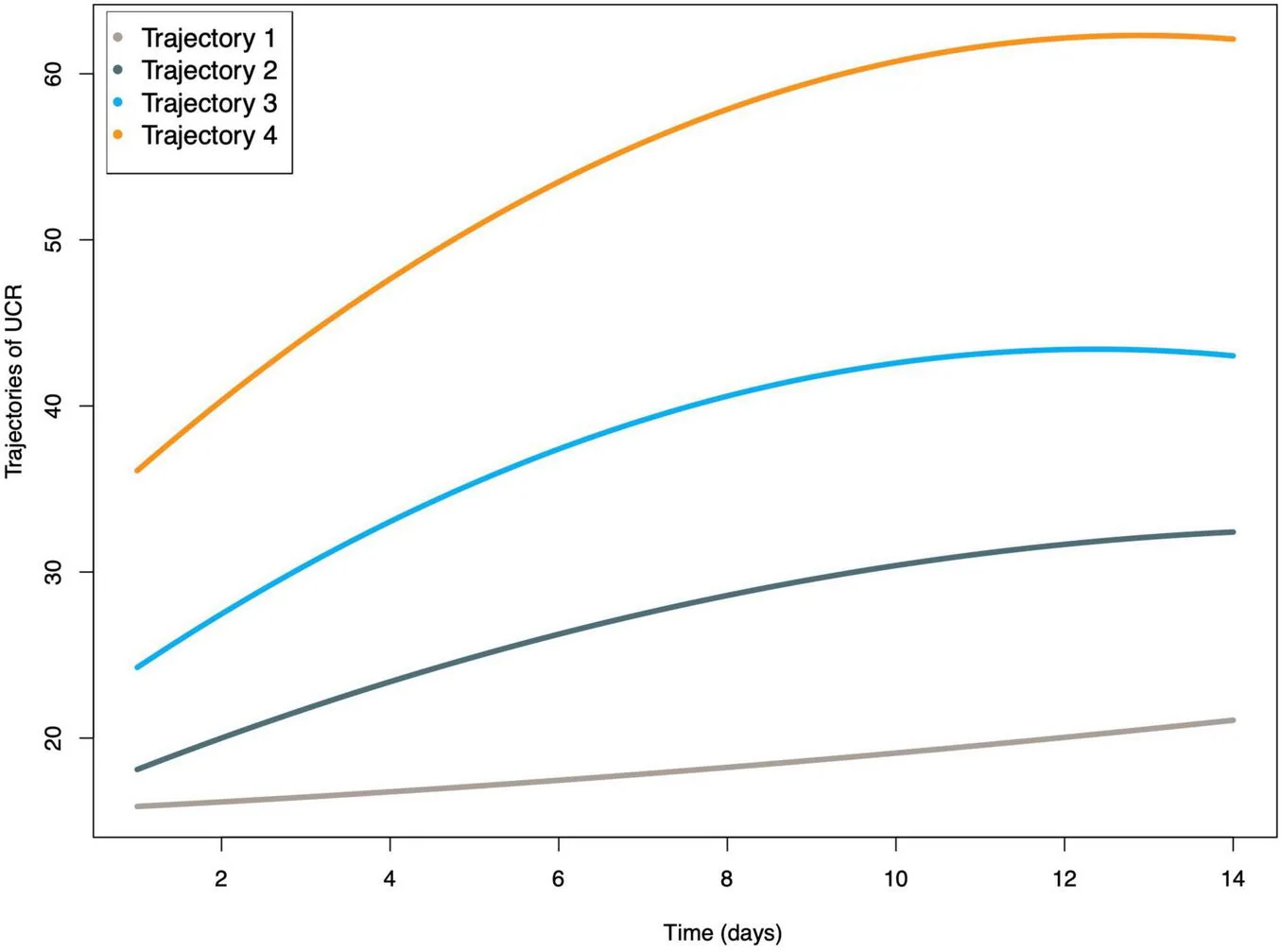
Urea-to-creatinine ratio (UCR) trajectories within the first 14 days after ICU admission.

The UCR trajectory groups are shown in Figure 2. Trajectory 1 (low-level, stable) was characterized by persistently low UCR over time. Trajectory 2 (low-level, slow increase) was characterized by a low UCR on ICU admission, with a gradual increase over time. Trajectory 3 (mid-level, slow increase) started with a mid-level UCR on ICU admission, followed by a mild rise over time. Trajectory 4 (high-level, rapid increase) had a noticeably elevated UCR on ICU admission that then showed a rapid increase. Daily BUN, creatinine, and urine output from day 1 to day 14 after ICU admission in different UCR trajectory groups are presented in Supplementary Figure 1.

Baseline characteristics of the total population and each UCR trajectory group are presented in Table 2. The median age of the patients was 65.8 years, and most patients were male (58.4%) and white (59.3%). A total of 1630 (94.9%) patients received invasive ventilation, 1674 (97.4%) patients developed sepsis during ICU stay. Compared with patients in the other trajectory groups, the patients in trajectory 1 group were younger and had the lowest Charlson Comorbidity Index and OASIS scores, as well as the lowest prevalence of sepsis and AKI. They exhibited the lowest proportion of invasive ventilation and norepinephrine administration. They also had highest prevalence of cerebrovascular disease.

**TABLE 2:** Clinical characteristics of the CCI patients with different UCR trajectory groups.

Variables	Overall (*n* = 1718)	Trajectory 1 (*n* = 391)	Trajectory 2 (*n* = 641)	Trajectory 3 (*n* = 507)	Trajectory 4 (*n* = 179)	*p*
Demographics						
Age (years)	65.8 [54.2, 74.9]	59.2 [46.2, 71.4]	65.5 [54.0, 74.6]	68.0 [57.2, 76.7]	68.4 [60.5, 76.1]	<0.001
Gender					<0.001	
Female	715 (41.6%)	141 (36.1%)	236 (36.8%)	237 (46.7%)	101 (56.4%)	
Male	1,003 (58.4%)	250 (63.9%)	405 (63.2%)	270 (53.3%)	78 (43.6%)	
BMI	28.6 (24.3, 33.7)	28.2 (23.9, 32.1)	28.8 (24.5, 34.2)	29.0 (24.6, 34.3)	28.1 (22.6, 32.7)	0.075
Race					0.004	
Asian	55 (3.2%)	18 (4.6%)	16 (2.5%)	17 (3.4%)	4 (2.2%)	
White	1,019 (59.3%)	217 (55.5%)	379 (59.1%)	304 (60.0%)	119 (66.5%)	
Black	161 (9.4%)	59 (15.1%)	59 (9.2%)	34 (6.7%)	9 (5.0%)	
Hispanic	64 (3.7%)	16 (4.1%)	21 (3.3%)	23 (4.5%)	4 (2.2%)	
Unknown	276 (16.1%)	54 (13.8%)	110 (17.2%)	86 (17.0%)	26 (14.5%)	
Other	143 (8.3%)	27 (6.9%)	56 (8.7%)	43 (8.5%)	17 (9.5%)	
Disease severity score						
GCS	15.0 [15.0, 15.0]	15.0 [15.0, 15.0]	15.0 [15.0, 15.0]	15.0 [15.0, 15.0]	15.0 [15.0, 15.0]	0.413
SOFA	1.0 [0.0, 3.0]	1.0 [0.0, 3.0]	1.0 [0.0, 3.0]	1.0 [0.0, 3.0]	1.0 [0.0, 3.0]	0.454
APS III	51.0 [39.0, 65.0]	50.0 [36.0, 65.0]	49.0 [37.0, 63.0]	52.0 [40.0, 65.0]	58.0 [44.0, 71.0]	<0.001
Charlson Comorbidity Index	5.0 [3.0, 7.0]	4.0 [2.0, 6.0]	5.0 [3.0, 7.0]	5.0 [3.0, 6.0]	5.0 [3.0, 7.0]	<0.001
OASIS	36.0 [31.0, 42.0]	35.0 [31.0, 41.0]	36.0 [31.0, 42.0]	36.0 [31.0, 42.0]	38.0 [34.0, 43.0]	<0.001
Comorbidities						
Hypertension	746 (43.4%)	158 (40.4%)	276 (43.1%)	235 (46.4%)	77 (43.0%)	0.353
Diabetes	498 (29.0%)	113 (28.9%)	190 (29.6%)	147 (29.0%)	48 (26.8%)	0.909
Congestive heart failure	490 (28.5%)	106 (27.1%)	169 (26.4%)	161 (31.8%)	54 (30.2%)	0.197
Liver disease	89 (5.2%)	23 (5.9%)	23 (3.6%)	29 (5.7%)	14 (7.8%)	0.088
Chronic pulmonary disease	501 (29.2%)	103 (26.3%)	175 (27.3%)	159 (31.4%)	64 (35.8%)	0.057
CKD	113 (6.6%)	38 (9.7%)	44 (6.9%)	23 (4.5%)	8 (4.5%)	0.011
Cerebrovascular disease	514 (29.9%)	134 (34.3%)	217 (33.9%)	129 (25.4%)	34 (19.0%)	<0.001
AKI	1,618 (94.2%)	361 (92.3%)	604 (94.2%)	484 (95.5%)	169 (94.4%)	0.262
Vital signs						
HR (bpm)	87 [75, 100]	87 [75, 100]	87 [76, 99]	86 [75, 99]	89 [76, 102]	0.737
RR (bpm)	20 [18, 23]	20 [17, 23]	20 [18, 23]	21 [18, 24]	21 [19, 25]	<0.001
SBP (mmHg)	115 [106, 127]	116 [106, 129]	115 [107, 128]	115 [106, 127]	115 [103, 126]	0.380
DBP (mmHg)	62 [56, 68]	63 [56, 71]	62 [57, 69]	61 [56, 67]	61 [57, 67]	0.162
MBP (mmHg)	78 [72, 85]	78 [72, 87]	79 [72, 85]	77 [72, 84]	76 [71, 84]	0.094
Laboratory indicators						
WBC (K/μL)	11.7 [8.3, 16.1]	11.9 [8.3, 16.1]	11.7 [8.5, 15.9]	11.5 [8.7, 16.5]	12.2 [7.8, 16.7]	0.926
Hemoglobin (g/dL)	11.3 [9.6, 12.9]	11.4 [9.6, 13.1]	11.3 [9.8, 13.0]	11.4 [9.6, 12.8]	10.7 [8.6, 12.5]	0.004
Hematocrit (%)	34.1 [29.0, 39.0]	34.0 [29.3, 39.2]	34.1 [29.3, 39.3]	34.8 [29.1, 38.9]	32.7 [27.1, 38.2]	0.109
RDW (%)	14.4 [13.4, 15.9]	14.3 [13.4, 15.6]	14.3 [13.2, 15.7]	14.5 [13.4, 16.0]	15.1 [13.8, 16.6]	<0.001
Platelet (K/μL)	200.0 [144.0, 271.0]	210.0 [155.0, 279.0]	198.0 [146.0, 265.0]	194.0 [133.0, 265.0]	202.0 [129.0, 279.0]	0.051
BUN (mg/dL)	18.0 [13.0, 29.0]	16.0 [11.0, 27.0]	17.0 [13.0, 26.0]	21.0 [15.0, 31.0]	25.0 [16.0, 39.0]	<0.001
Creatinine (mg/dL)	0.9 [0.7, 1.3]	1.1 [0.8, 1.4]	1.0 [0.7, 1.3]	0.9 [0.7, 1.2]	0.8 [0.6, 1.1]	<0.001
Anion gap (mmol/L)	14.0 [12.0, 16.0]	14.0 [12.0, 17.0]	14.0 [12.0, 16.0]	14.0 [12.0, 17.0]	13.0 [11.0, 16.0]	<0.001
Bicarbonate (mmol/L)	23.0 [20.0, 26.0]	22.0 [19.0, 25.0]	23.0 [20.0, 25.0]	23.0 [20.0, 26.0]	24.0 [21.0, 28.0]	<0.001
Sodium (mEq/L)	138.0 [135.0, 141.0]	138.0 [135.0, 141.0]	138.0 [136.0, 141.0]	138.0 [135.0, 141.0]	139.0 [134.0, 142.0]	0.680
Chloride (mEq/L)	104.0 [100.0, 108.0]	104.0 [100.0, 108.0]	104.0 [100.0, 108.0]	103.0 [99.0, 107.0]	103.0 [100.0, 107.0]	0.008
Potassium (mEq/L)	4.0 [3.6, 4.4]	3.9 [3.5, 4.4]	4.0 [3.6, 4.4]	4.0 [3.7, 4.4]	4.1 [3.7, 4.5]	0.061
Calcium (mg/dL)	8.3 [7.8, 8.8]	8.2 [7.6, 8.7]	8.3 [7.8, 8.8]	8.4 [7.8, 8.8]	8.3 [7.9, 8.9]	0.083
Glucose (mg/dL)	137.5 [112.0, 175.0]	131.0 [109.0, 175.0]	138.0 [114.0, 174.0]	140.0 [112.0, 179.0]	142.0 [107.0, 176.0]	0.383
AST (IU/L)	48.0 [27.0, 91.0]	48.0 [27.0, 103.0]	51.0 [28.0, 95.1]	47.0 [26.0, 86.0]	39.0 [25.0, 73.3]	0.039
ALT (IU/L)	33.0 [18.0, 65.0]	32.0 [18.0, 67.0]	33.7 [18.0, 67.0]	33.0 [19.0, 63.0]	29.0 [17.0, 61.0]	0.614
DBIL (μmol/L)	0.7 [0.4, 1.2]	0.8 [0.4, 1.3]	0.7 [0.4, 1.1]	0.7 [0.4, 1.1]	0.6 [0.4, 1.3]	0.546
PT (sec)	13.9 [12.4, 16.4]	13.8 [12.5, 16.0]	13.7 [12.3, 16.4]	13.9 [12.4, 16.6]	14.0 [12.4, 17.0]	0.579
APTT (sec)	30.4 [27.1, 37.0]	30.1 [26.7, 37.3]	30.2 [27.1, 36.9]	30.8 [27.1, 37.3]	30.8 [28.1, 38.0]	0.561
INR	1.3 [1.1, 1.5]	1.3 [1.1, 1.5]	1.3 [1.1, 1.5]	1.3 [1.1, 1.5]	1.3 [1.1, 1.5]	0.596
PaO_2_ (mmHg)	123.0 [83.0, 194.0]	142.0 [87.0, 207.0]	132.0 [83.0, 216.0]	117.0 [82.0, 166.0]	105.0 [77.0, 157.5]	<0.001
PaCO_2_ (mmHg)	42.0 [35.0, 49.0]	41.0 [34.0, 46.0]	41.0 [35.0, 48.0]	42.0 [35.0, 51.0]	43.9 [36.0, 54.0]	<0.001
P/F ratio	221.0 [133.3, 324.0]	230.9 [155.0, 328.0]	225.0 [132.0, 337.0]	209.1 [126.7, 313.0]	209.0 [118.8, 278.0]	0.008
Lactate (mmol/L)	1.6 [1.1, 2.5]	1.7 [1.1, 2.7]	1.6 [1.1, 2.5]	1.6 [1.1, 2.4]	1.6 [1.1, 2.3]	0.712
Treatment						
Albumin infusion	678 (39.5%)	171 (43.7%)	250 (39.0%)	187 (36.9%)	70 (39.1%)	0.216
Norepinephrine	648 (37.7%)	131 (33.5%)	233 (36.3%)	205 (40.4%)	79 (44.1%)	0.042
Epinephrine	554 (32.2%)	121 (30.9%)	224 (34.9%)	160 (31.6%)	49 (27.4%)	0.213
ICU course characteristics						
Sepsis	1,674 (97.4%)	370 (94.6%)	630 (98.3%)	499 (98.4%)	175 (97.8%)	0.003
Invasive ventilation	1,630 (94.9%)	361 (92.3%)	606 (94.5%)	488 (96.3%)	175 (97.8%)	0.015
Clinical outcomes						
Hospital LOS (days)	26.4 [20.6, 35.8]	26.4 [20.9, 34.8]	27.0 [20.8, 37.3]	26.1 [21.4, 35.5]	23.9 [18.4, 32.0]	0.010
ICU LOS (days)	19.3 [16.2, 25.0]	18.7 [15.7, 22.9]	19.7 [16.3, 25.6]	20.1 [16.7, 25.7]	18.7 [16.4, 24.4]	<0.001
Hospital mortality	350 (20.4%)	48 (12.3%)	123 (19.2%)	117 (23.1%)	62 (34.6%)	<0.001
ICU mortality	243 (14.1%)	27 (6.9%)	85 (13.3%)	83 (16.4%)	48 (26.8%)	<0.001
28-day mortality	374 (21.8%)	49 (12.5%)	135 (21.1%)	117 (23.1%)	73 (40.8%)	<0.001
90-day mortality	521 (30.3%)	75 (19.2%)	191 (29.8%)	163 (32.1%)	92 (51.4%)	<0.001
365-day mortality	675 (39.3%)	106 (27.1%)	238 (37.1%)	222 (43.8%)	109 (60.9%)	<0.001

BMI, body mass index; GCS, Glasgow Coma Scale; SOFA, Sequential Organ Failure Assessment; APS III, Acute Physiology III; OASIS, Oxford Acute Severity of Illness Score; CKD, chronic kidney disease; AKI, acute kidney injury; HR, heart rate; RR, respiratory rate; SBP, systolic blood pressure; DBP, diastolic blood pressure; MBP, mean blood pressure; WBC, white blood cell; RDW, red cell distribution width; BUN, blood urea nitrogen; AST, aspartate aminotransferase; ALT, alanine aminotransferase; DBIL, direct bilirubin; PT, prothrombin time; APTT, activated partial thromboplastin time; INR, international normalized ratio; PaO_2_, partial pressure of oxygen; PaCO_2_, partial pressure of carbon dioxide; P/F Ratio, ratio of partial pressure of oxygen to fraction of inspired oxygen; LOS, length of stay; ICU, intensive care unit.

### Survival analysis

**FIGURE 3:**
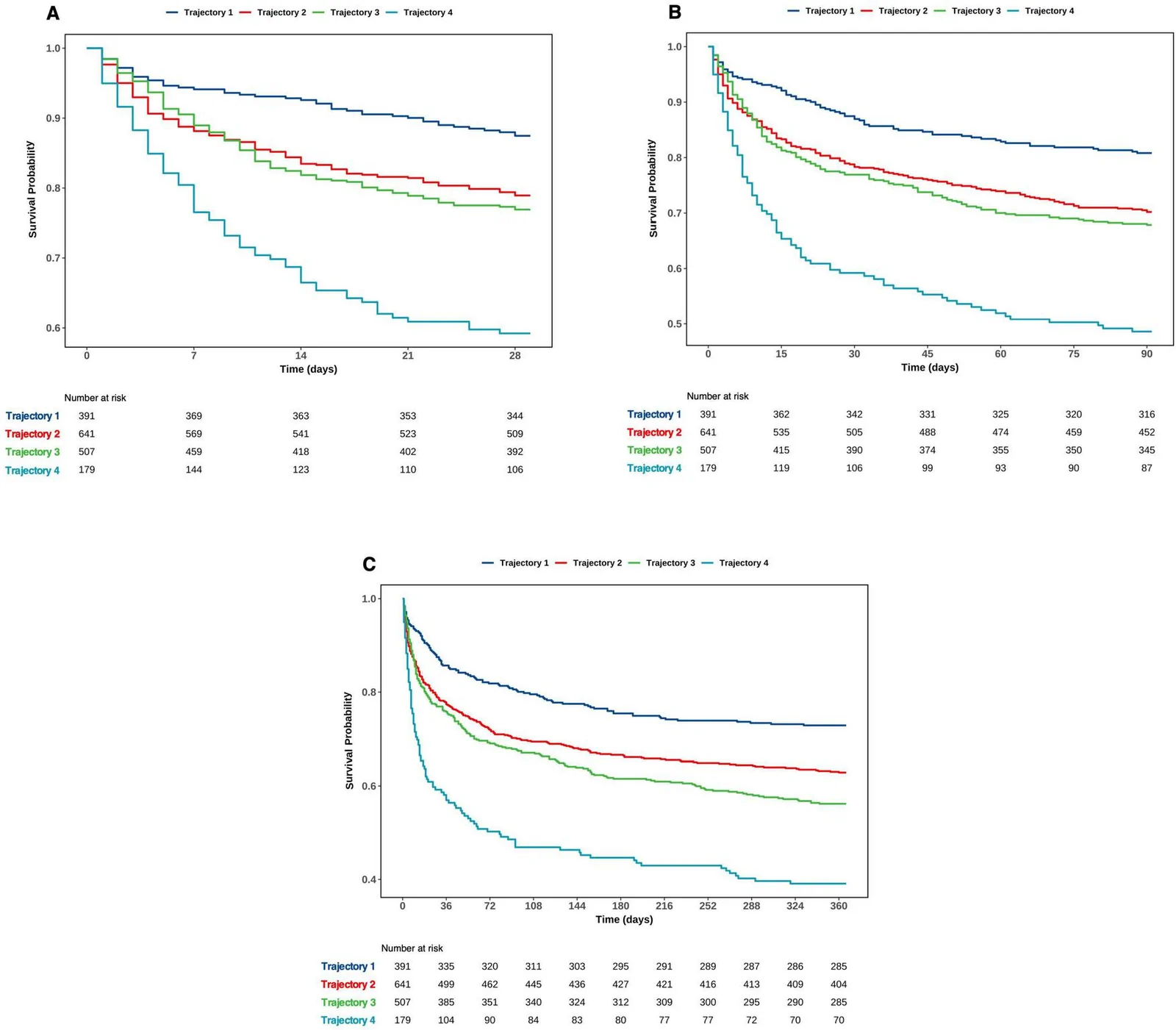
Kaplan–Meier survival analysis for mortality among the UCR trajectory groups. Kaplan–Meier curves of 28-day **(A)**, 90-day **(B)** and 365-day **(C)** mortality stratified by UCR trajectories.

Trajectory 4 group had the highest 28-day, 90-day, and 365-day mortality, as well as the ICU and hospital mortality, while trajectory 1 group had the lowest mortality. Kaplan–Meier analysis revealed significant differences in 28-day, 90-day, and 365-day mortality among the UCR trajectory groups (Figure 3). The cumulative 28-day, 90-day, and 365-day mortality was significantly and progressively increased from trajectory 1 to trajectory 4 group.

### Association between UCR trajectories and clinical outcomes

To further investigate the independent effects of UCR trajectory groups on mortality, we conducted two Cox proportional hazards models (Table 3). The crude model was unadjusted for any confounders. Model I adjusted for nine confounding variables identified by the DAGs (Supplementary Figure 2), which included gender, age, BMI, congestive heart failure, liver disease, CKD, AKI, sepsis, and albumin infusion. In the crude model, compared to the reference group (trajectory 1), the 28-day, 90-day and 365-day mortality risks were significantly higher in the other trajectory groups. Model I showed a similar trend, with 28-day, 90-day, and 365-day mortality risks remaining elevated in trajectory 2–4 groups compared to trajectory 1 group.

**TABLE 3:** Multivariable Cox regression analysis for different UCR trajectory groups and 28-day, 90-day and 365-day mortality.

Outcome	Crude model		Model I	
	HR (95% CI)	*p*	HR (95% CI)	*p*
28-day mortality				
Trajectory 1	Reference		Reference	
Trajectory 2	1.79 (1.29–2.48)	<0.001	1.60 (1.15–2.22)	0.005
Trajectory 3	1.96 (1.40–2.73)	<0.001	1.63 (1.16–2.29)	0.005
Trajectory 4	3.95 (2.75–5.67)	<0.001	3.15 (2.17–4.56)	<0.001
90-day mortality				
Trajectory 1	Reference		Reference	
Trajectory 2	1.68 (1.28–2.19)	<0.001	1.51 (1.15–1.97)	0.003
Trajectory 3	1.83 (1.39–2.40)	<0.001	1.53 (1.15–2.02)	0.003
Trajectory 4	3.48 (2.57–4.72)	<0.001	2.81 (2.05–3.84)	<0.001
365-day mortality				
Trajectory 1	Reference		Reference	
Trajectory 2	1.50 (1.19–1.88)	<0.001	1.34 (1.06–1.68)	0.014
Trajectory 3	1.81 (1.44–2.28)	<0.001	1.50 (1.18–1.90)	<0.001
Trajectory 4	3.11 (2.38–4.06)	<0.001	2.49 (1.89–3.27)	<0.001

HR, hazard ratio; CI, confidence interval. Crude model: Unadjusted; Model I: Adjusted for gender, age, BMI, congestive heart failure, liver disease, CKD, AKI, sepsis, and albumin infusion.

### Subgroup analysis

**FIGURE 4:**
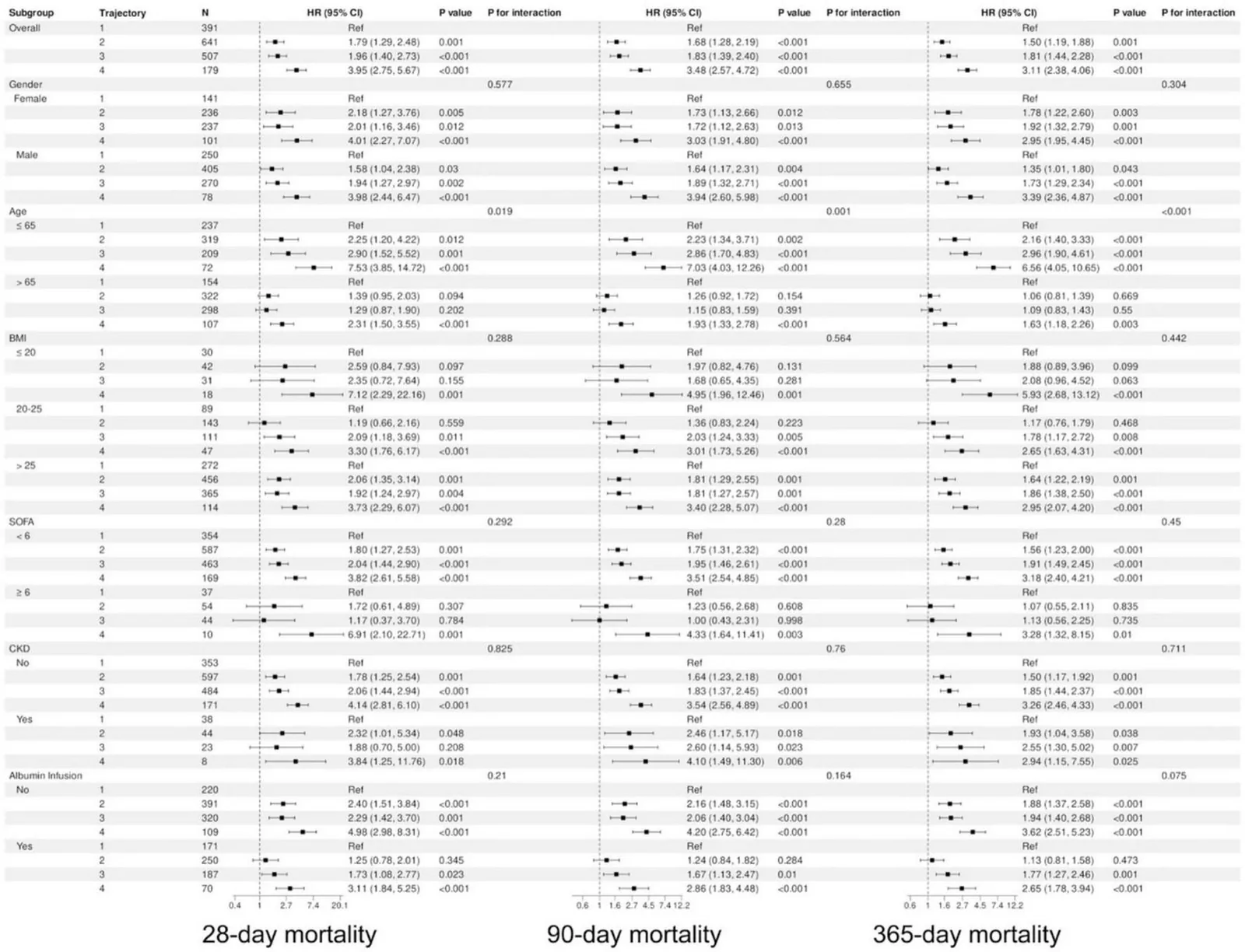
Subgroup analysis of the association between UCR trajectories and mortality.

Subgroup analyses were conducted to evaluate the robustness of the relationship between UCR trajectories and 28-day, 90-day, and 365-day mortality across various subgroups (stratified by gender, age, BMI, SOFA score, CKD, and albumin infusion). The results are shown in Figure 4. To minimize potential false-positive findings, we implemented a Bonferroni correction to adjust for multiple comparisons. No significant interactions were found between UCR trajectory groups and gender, BMI, SOFA score, CKD or albumin infusion (P for interaction > 0.05). Notably, there was a statistically significant interaction between age and UCR trajectory groups on 28-day, 90-day and 365-day mortality (P for interaction = 0.019, 0.001 and <0.001, respectively). Among patients aged ≤ 65 years, 28-day, 90-day and 365-day mortality risk increased more steeply across UCR trajectories; in patients >65 years, the association was present but less pronounced.

### Sensitivity analysis

In the sensitivity analysis, we included 659 CCI patients with admission creatinine > 4 mg/dL and/or RRT to assess the robustness of the UCR–mortality association in patients with severe renal dysfunction. Using the same model-fit criteria as in the primary analysis, a three-group trajectory model was selected (Supplementary Figure 3). The cumulative 28-day, 90-day, and 365-day mortality was highest in trajectory 3, which represented the high-level, rapidly-increasing UCR phenotype (Supplementary Figure 4). In the multivariable Cox proportional hazards model, this high-level, rapidly-increasing trajectory remained associated with higher 90-day and 365-day mortality risk compared with trajectory 1, after adjusting for multiple confounding factors (Supplementary Table 2).

## Discussion

In this retrospective cohort study, we used GBTM to investigate the UCR trajectory phenotypes among adult CCI patients and demonstrated their association with clinical outcomes. Four distinct UCR trajectory groups were identified: trajectory 1 (low-level, stable), trajectory 2 (low-level, slow increase), trajectory 3 (mid-level, slow increase) and trajectory 4 (high-level, rapid increase) and these patterns were strongly correlated with mortality risk. Patients in the high-level UCR upon ICU admission and rapidly increasing trajectory group had the worst survival (highest 28-day, 90-day, and 365-day mortality), whereas those in the low-level, stable UCR group had the most favorable outcomes. Intermediate trajectories fell in between, suggesting a gradation of risk that corresponds to the severity and persistence of catabolic stress. Notably, these associations remained significant even after adjusting for confounders, indicating that the dynamic trend of UCR in the first 2 weeks of ICU stay was an independent indicator of prognosis in CCI patients. In practical terms, a persistently elevated and rising UCR trajectory appears to be a warning sign of poor outcome in CCI patients, while a lower, stable trajectory portends a relatively better prognosis.

These findings support the concept that a profoundly dysregulated catabolic state underlies poor outcomes in CCI patients (11, 14). CCI is often characterized by ongoing inflammation, immunosuppression, and extensive protein catabolism despite adequate support (29). A previous study by Puthucheary et al. has demonstrated that rapid skeletal muscle wasting within the first week of ICU admission despite nutritional therapy (16), highlighting the dominance of catabolic drive over anabolic efforts. This muscle loss, evidenced by reductions in muscle cross-sectional area (CSA) and muscle fiber content has been linked more to intrinsic metabolic disturbances than to inadequate substrate delivery (15, 30). In other words, patients who progress to CCI often experience refractory catabolism: their bodies break down protein and muscle at a high rate, which conventional feeding cannot fully counteract. The UCR reflects complex metabolic and physiological processes. Urea levels may rise with increased protein breakdown, whereas creatinine production can decline with reduced muscle mass (31, 32). However, UCR is also influenced by renal function, hydration status, gastrointestinal bleeding, and nutritional intake. Therefore, a high-level rapid-increase UCR phenotype likely reflects a state of heightened metabolic stress and physiological deterioration, rather than isolated muscle proteolysis alone. In contrast, a low-level stable phenotype may represent patients with better metabolic control, fewer catabolic triggers, or earlier resolution of systemic inflammation. Low-/mid-level slow-increase phenotypes plausibly index evolving catabolism driven by intercurrent complications, cumulative organ dysfunction, or suboptimal rehabilitation and nutrition. Our identification of a high-and-rising UCR subgroup aligns with this understanding; it represents patients trapped in a catabolic spiral, which in turn contributes to organ dysfunction, weakness, and mortality. Recent critical-care literature emphasizes that UCR should be interpreted as a composite metabolic-renal signal rather than a pure marker of muscle proteolysis, because exogenous protein delivery, renal handling of urea, hydration status, gastrointestinal bleeding, and renal replacement therapy all modulate the ratio (33, 34). Accordingly, the high-and-rising UCR phenotype is best interpreted as a composite metabolic-renal phenotype reflecting accelerated protein catabolism, reduced muscle mass, hypovolemia, sepsis-related metabolic stress, and renal handling of urea, rather than as a marker of any single mechanism.

Notably, we observed a significant interaction by age: the gradient of risk across UCR trajectories was steeper among patients ≤ 65 years, while the association persisted but was attenuated in those >65 years. The stronger risk gradient across trajectory groups in patients ≤ 65 years could be due to lower baseline sarcopenia (yielding greater relative signal in UCR changes) and higher physiological reserve that makes deviations more prognostically informative; in older adults, pre-existing sarcopenia and comorbidity may “saturate” catabolic risk (35), attenuating incremental effects, an interpretation that requires prospective confirmation.

The clinical implications of monitoring UCR trajectories in the ICU are considerable. UCR is an inexpensive and readily obtainable metric from routine blood tests, which makes it an attractive biomarker for bedside use (17). Recent studies have provided evidence for UCR to be applied as a measure of persistent critical illness (19). In a cohort study of 1173 critically ill polytrauma patients, Haines et al. demonstrated that an elevated UCR is associated with increased muscle wasting and prolonged ICU stays, highlighting its potential as a biochemical signature of catabolism. Our results suggest that identifying a patient’s UCR trajectory early in the ICU stay could provide clinicians with a real-time window into the patient’s metabolic state. Those who manifest a high and rising UCR (such as our trajectory 4 phenotype) can be recognized as being in a profound catabolic condition and at significantly elevated risk of adverse outcomes. In practice, this could prompt earlier and more aggressive interventions aimed at mitigating catabolism and supporting recovery. For example, clinicians might intensify nutritional support (ensuring adequate protein and caloric intake), individualized rehabilitation and mobilization or consider anabolic adjunct therapies in a patient whose UCR trajectory is trending sharply upward despite standard care. At the same time, because exogenous amino-acid or protein delivery may itself increase ureagenesis and raise UCR independently of endogenous catabolism, nutritional decisions should be individualized and interpreted together with renal function, the delivered protein dose, and the overall clinical context rather than guided by UCR alone. Additionally, recognition of a worsening UCR trajectory might trigger evaluations for underlying treatable drivers of catabolism, such as uncontrolled infection or unaddressed endocrine imbalances, and heighten surveillance for complications. In addition to guiding therapy, UCR trajectory phenotypes could inform prognostication and care planning. Patients in a persistently high UCR trajectory may have a limited likelihood of rapid recovery, which is valuable information for multi-disciplinary care teams and family discussions regarding goals of care, rehabilitation needs, or possible transfer to long-term acute care facilities. Overall, incorporating UCR trajectory monitoring into critical care practice could enhance our ability to phenotype CCI patients at the bedside. It offers a practical biomarker to flag the development of CCI. However, such monitoring must be coupled with action: the true benefit will be realized if clinicians respond to adverse trajectory with tailored interventions. Our study lays the foundation by establishing the prognostic significance of UCR trends in CCI populations. Future prospective multicenter studies should validate these UCR trajectory phenotypes across different ICU populations and settings, and explore whether interventions for patients with high-risk UCR trajectories can be beneficial. This caution is supported by recent evidence that higher exogenous protein intake itself raises UCR independently of endogenous catabolism (34); UCR-guided nutrition titration must therefore integrate the delivered protein dose, renal function, and the overall clinical context, rather than rely on UCR alone.

Our study adds novel insights to the field by capturing the longitudinal trajectories of a catabolic marker in CCI patients. Previous research has mostly examined UCR at single time points or average values (36–38), whereas we employed GBTM to uncover heterogeneity in how UCR evolves over time. Unlike single time-point measurements, trajectory analysis captures both the absolute level and the temporal slope of biomarker evolution, thereby reflecting the dynamic progression of metabolic stress. GBTM adds value by providing a patient-level phenotypic classification that may facilitate clinical communication and future subgroup-targeted intervention studies. The four trajectory groups identified by GBTM differing in both baseline UCR magnitude and rate of change over 14 days, highlighting patient heterogeneity that would not be apparent from a single UCR measurement at ICU admission. The discovery of four trajectory phenotypes is important: it shows that CCI patients are not a homogeneous group metabolically. This heterogeneity had not been well characterized before and represents a new way to phenotype CCI. By linking each trajectory with both short-term and longer-term outcomes, we provide evidence that the time course of catabolism (not just a one-time measurement) carries prognostic significance in CCI patients. Furthermore, the large sample size and strict control of confounding factors further enhance the credibility of our conclusions. We utilized a large, high-quality dataset (MIMIC-IV) encompassing a diverse cohort of 1718 CCI patients. The substantial sample size provided adequate power and confidence in the stability of the trajectory groups identified and the associations observed. By using DAGs to select confounders, we avoided overfitting and controlled for a comprehensive range of factors (demographics, comorbidities, severity scores, treatments) that could influence both UCR and outcomes. Importantly, the day-1 to day-14 trends of BUN, serum creatinine, and urine output across UCR trajectory groups (Supplementary Figure 1) showed that trajectory 4 was characterized by higher BUN combined with relatively lower creatinine, a pattern more consistent with increased ureagenesis and reduced creatinine generation than with isolated renal failure. This contextual information supports a composite metabolic-renal interpretation of the UCR trajectories rather than a pure renal interpretation.

However, there are several limitations in our study. First, our data were derived from a single hospital database (MIMIC-IV dataset). While this database includes a broad mix of medical, surgical, and trauma patients, the results may not be fully generalizable to all ICU settings. Second, although we adjusted for many potential confounders using DAGs to inform our model, residual confounding from unmeasured variables is possible. Factors such as the exact nutritional strategy, variations in fluid balance, or unrecorded comorbid conditions could influence UCR and outcomes but were not fully captured. In particular, daily protein dose and amino-acid delivery could not be reliably modeled as time-dependent covariates because nutrition records in MIMIC-IV have variable granularity and protein dose was missing in a substantial proportion of patient-days. Similarly, inflammatory and nutritional markers such as CRP, IL-6, serum albumin, and prealbumin could not be incorporated into the primary models because of unavailability or excessive missingness. Third, we focused on UCR measurements during the first 14 days of ICU stay (by definition, all patients were in ICU for at least 14 days); however, some patients remained critically ill far beyond this period. We did not examine whether UCR trajectories beyond 2 weeks continue to diverge or whether patients could transition between trajectory groups with longer ICU courses. Fourth, as in previous works (19, 22, 23), we excluded patients who were treated with RRT or had creatinine > 4 mg/dL. However, patients who need RRT are usually sicker, hence it is likely that a higher proportion of them will suffer from CCI. These exclusions limit the generalizability of this study. In such patients, using UCR as a metabolic signature may be challenging and alternative markers might be needed. Fifth, UCR is influenced by multiple factors beyond protein catabolism, including renal function, hydration status, dietary protein intake, and gastrointestinal blood absorption (18). While we excluded patients with severe kidney dysfunction (creatinine > 4 mg/dL), those receiving RRT, and those with gastrointestinal bleeding at ICU admission, these exclusions do not fully control for hypovolemia or variations in dietary protein intake, which were not systematically recorded in the MIMIC-IV database. Accordingly, UCR should be interpreted as a composite metabolic indicator rather than a specific marker of catabolism, and clinicians should consider the full clinical context when interpreting UCR values. Finally, as an observational study, we can only demonstrate association, not causation. We cannot conclude that changing CCI patient’s UCR trajectory (for example, through an intervention) will necessarily improve outcomes; that remains to be tested in future studies. Additionally, the covariates included in our Cox proportional hazards model were not all measured at the same time point: most baseline variables were recorded at ICU admission, while sepsis and invasive ventilation reflect events occurring across the 14-day ICU window. This temporal inconsistency is a limitation of our analytical approach. Future studies using time-varying covariate models (e.g., time-varying Cox models) would provide a more rigorous assessment of the independent association between UCR trajectories and mortality. In addition, recent evidence has shown that continuous renal replacement therapy itself modulates UCR, patients with a high baseline UCR (≥75 mg/dL:mg/dL) experience a marked reduction in UCR after CRRT initiation, falling below the 75 mg/dL:mg/dL threshold by day 3 of therapy, while patients with low baseline UCR show an attenuated increase (33). UCR therefore cannot be directly extrapolated to the RRT setting, and dedicated prospective validation is required before applying UCR-based phenotyping to patients receiving continuous renal support.

## Conclusion

In conclusion, our study provides evidence that longitudinal UCR trajectories serve as a meaningful biomarker reflecting metabolic stress and overall physiological burden in CCI patients and are strongly associated with short- and long-term mortality. Monitoring the UCR trend captures dynamic illness progression and identifies heterogeneous risk phenotypes beyond single time-point measurements. Future prospective studies are needed to validate these findings and to explore whether recognition of adverse metabolic trajectories can inform risk stratification and guide personalized strategies of CCI.

## Data Availability

The original contributions presented in this study are included in this article/Supplementary material, further inquiries can be directed to the corresponding authors.

## Footnotes

1
www.r-project.org
