# Correction: Association between urea-to-creatinine ratio trajectories and clinical outcomes in chronic critical illness patients: a retrospective cohort study

**DOI:** 10.3389/fnut.2026.1946337

**Published:** 2026-08-05

**Authors:** Frontiers Production Office

**Affiliations:** Frontiers Media SA, Lausanne, Switzerland

**Keywords:** chronic critical illness, group-based trajectory model, MIMIC-IV database, prognosis, urea-to-creatinine ratio

There was a mistake in Figure 4 as published. The image displayed as Figure 4 was inadvertently published and not related to this study. The corrected Figure 4 appears below.

**Figure 4:**
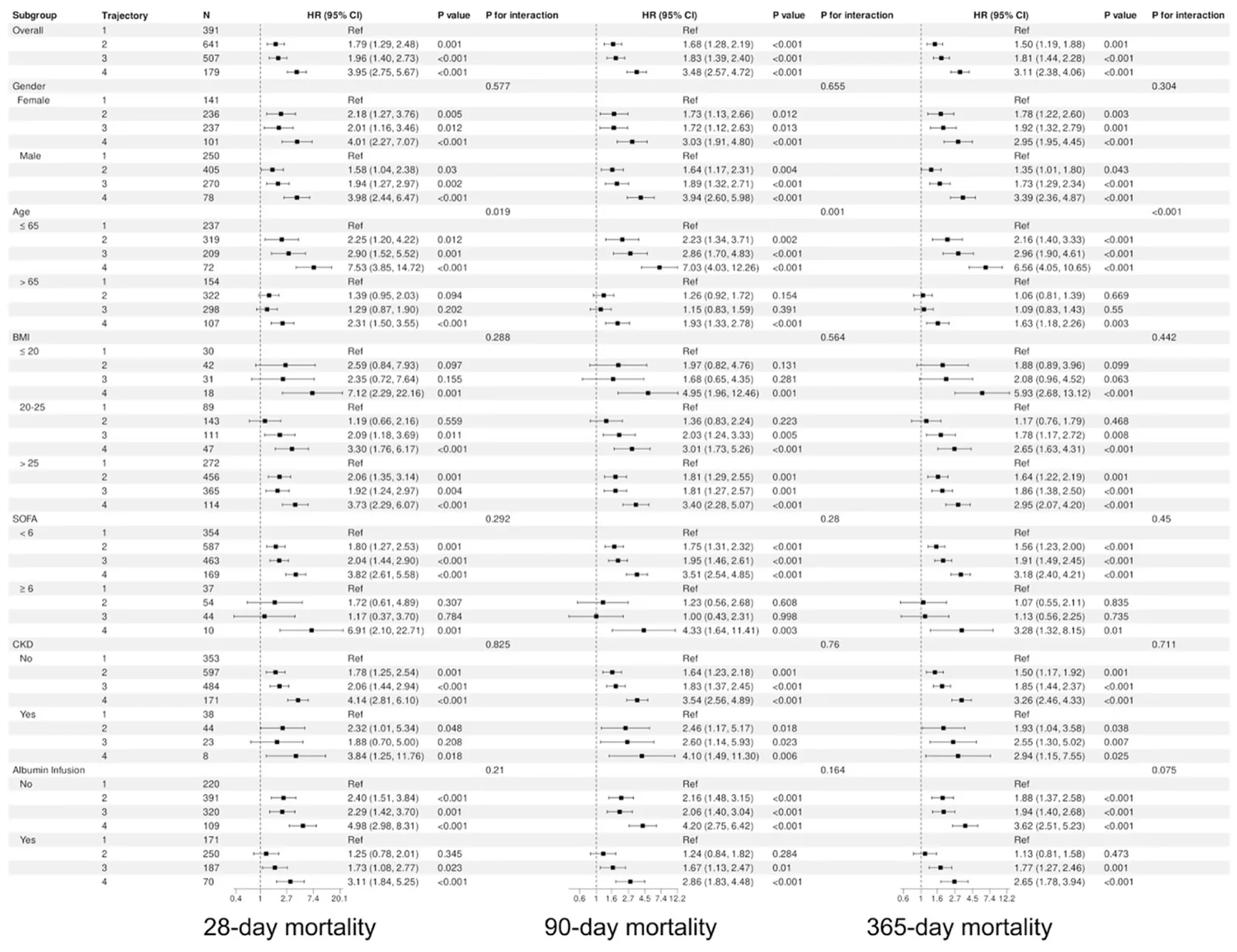
Subgroup analysis of the association between UCR trajectories and mortality.

The original version of this article has been updated.

## Generative AI statement

Any alternative text (alt text) provided alongside figures in this article has been generated by Frontiers with the support of artificial intelligence and reasonable efforts have been made to ensure accuracy, including review by the authors wherever possible. If you identify any issues, please contact us.

