# Supplementary material for: Association between urea-to-creatinine ratio trajectories and clinical outcomes in chronic critical illness patients: a retrospective cohort study
Source: Front Nutr. 2026 Jul 6;13:1776277. doi: 10.3389/fnut.2026.1776277 (PMC13381204; doi:10.3389/fnut.2026.1776277)
Supplement: Supplementary file 1 [file Data_Sheet_1.pdf]

Association between urea-to-creatinine ratio trajectories and clinical outcomes in chronic critical illness patients: a retrospective cohort study

Supplementary Material

Table S1: Missing rate for demographics and clinical variables extracted from the database.

Variable	Number of missing	Percent of missing (%)
Age	0	0
Gender	0	0
BMI	0	0
Race	0	0
GCS	0	0
SOFA	0	0
APS III	0	0
Charlson Comorbidity Index	0	0
OASIS	0	0
Hypertension	0	0
Diabetes	0	0
Congestive heart failure	0	0
Liver disease	0	0
Chronic pulmonary disease	0	0
Renal disease	0	0
Cerebrovascular disease	0	0
Sepsis	0	0
AKI	0	0
P/F ratio	126	7.3
Lactate	171	10.0
Hematocrit	0	0
RDW	0	0
Platelet	0	0

WBC	0	0
Creatinine	0	0
BUN	0	0
Anion gap	0	0
Calcium	0	0
PT	22	1.3
APTT	21	1.2
INR	22	1.3
ALT	258	15.0
AST	252	14.7
DBIL	269	15.7
Chloride	0	0
Glucose	0	0
Potassium	0	0
Bicarbonate	0	0
Hemoglobin	0	0
PaO_2	79	4.6
$PaCO_2$	79	4.6

BMI, Body Mass Index; GCS, Glasgow Coma Scale; SOFA, Sequential Organ Failure Assessment; APS III, Acute Physiology III; OASIS, Oxford Acute Severity of Illness Score; AKI, Acute Kidney Injury; WBC, White Blood Cell; RDW, Red Cell Distribution Width; BUN, Blood Urea Nitrogen; AST, Aspartate Aminotransferase; ALT, Alanine Aminotransferase; DBIL, Direct Bilirubin; PT, Prothrombin Time; APTT, Activated Partial Thromboplastin Time; INR, International Normalized Ratio; PaO2, partial pressure of oxygen; PaCO2, partial pressure of carbon dioxide; P/F Ratio, Ratio of Partial Pressure of Oxygen to Fraction of Inspired Oxygen.

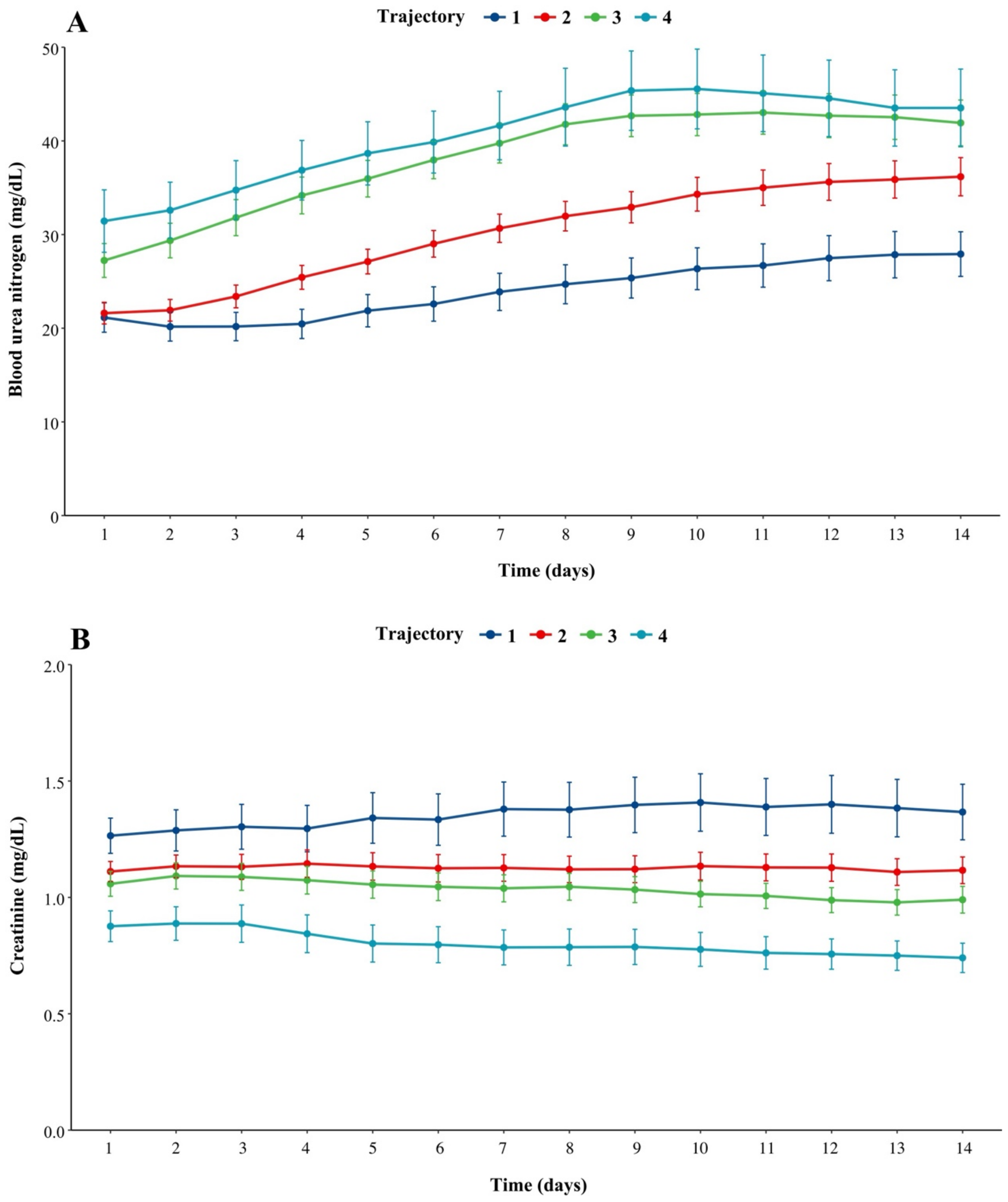

A
Trajectory 1 2 3 4
Blood urea nitrogen (mg/dL)
Time (days)
B
Trajectory 1 2 3 4
Creatinine (mg/dL)
Time (days)

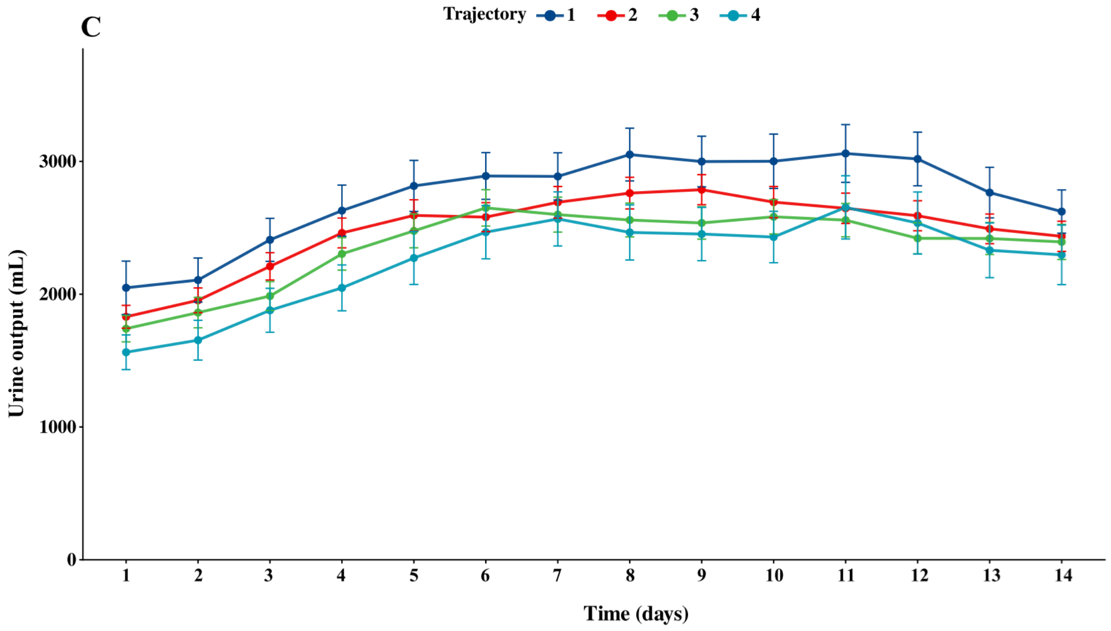

Figure S1: Line charts of daily BUN (A), creatinine (B), and urine output (C) from day 1 to day 14 after ICU admission in different UCR trajectory groups.

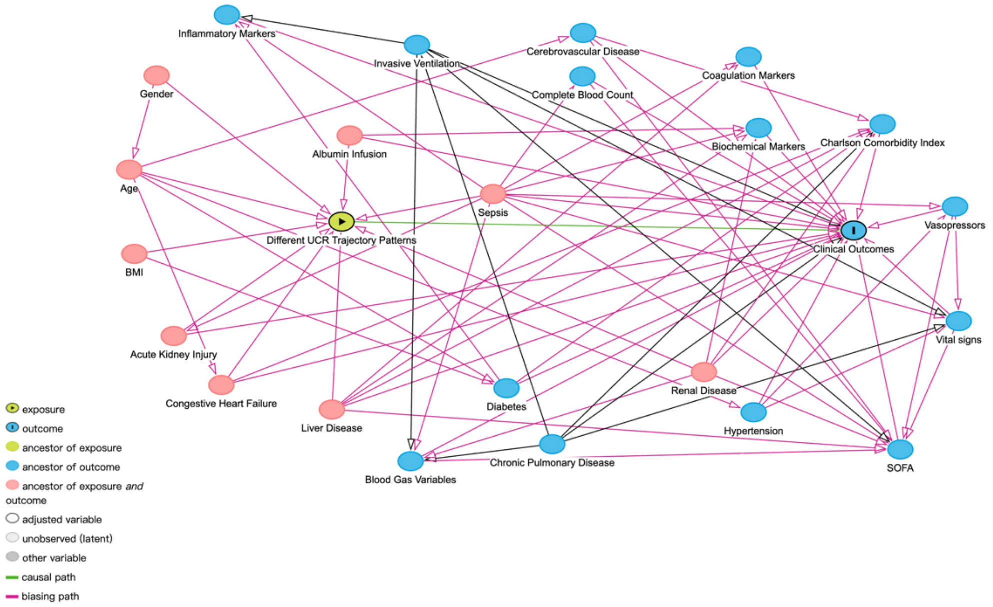

Figure S2: Directed acyclic graph (DAG) illustrating the potential effects of confounding covariates on the relationship between different UCR trajectory patterns and clinical outcomes.

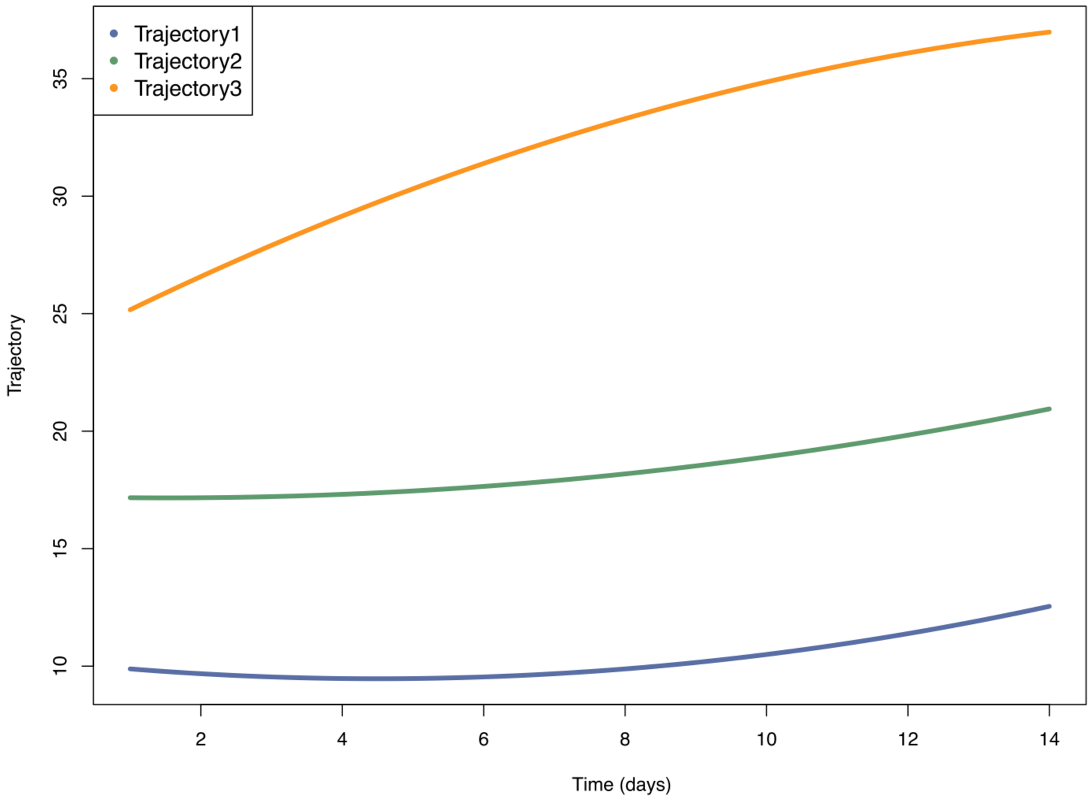

Figure S3: UCR trajectories within the first 14 days after ICU admission in patients with admission creatinine >4 mg/dL and/or RRT.

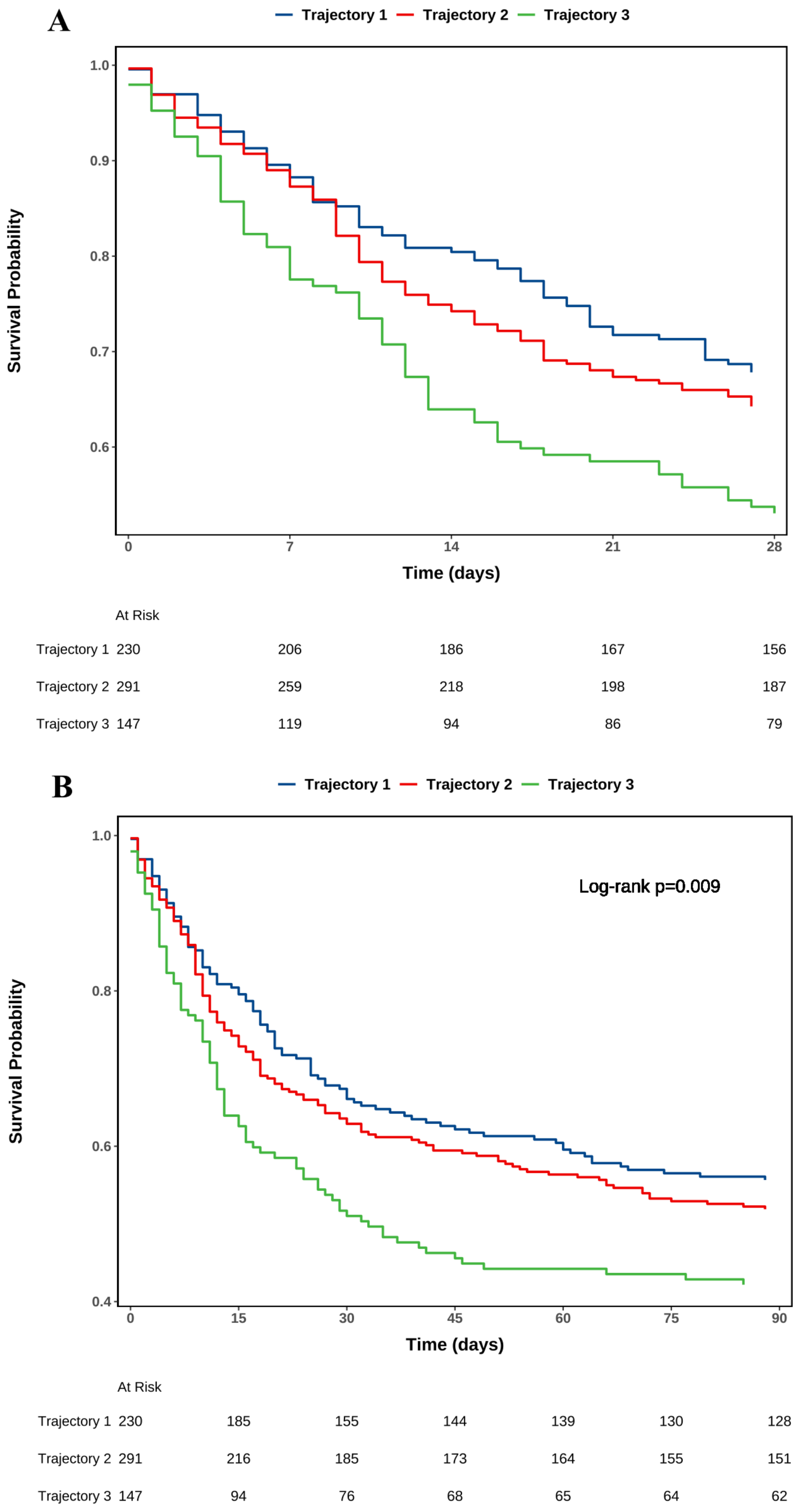
A
Trajectory 1
Trajectory 2
Trajectory 3
Survival Probability
1.0
0.9
0.8
0.7
0.6
0
7
14
21
28
Time (days)
At Risk
Trajectory 1 230 206 186 167 156
Trajectory 2 291 259 218 198 187
Trajectory 3 147 119 94 86 79
B
Trajectory 1
Trajectory 2
Trajectory 3
Log-rank p=0.009
Survival Probability
1.0
0.8
0.6
0.4
0
15
30
45
60
75
90
Time (days)
At Risk
Trajectory 1 230 185 155 144 139 130 128
Trajectory 2 291 216 185 173 164 155 151
Trajectory 3 147 94 76 68 65 64 62

C

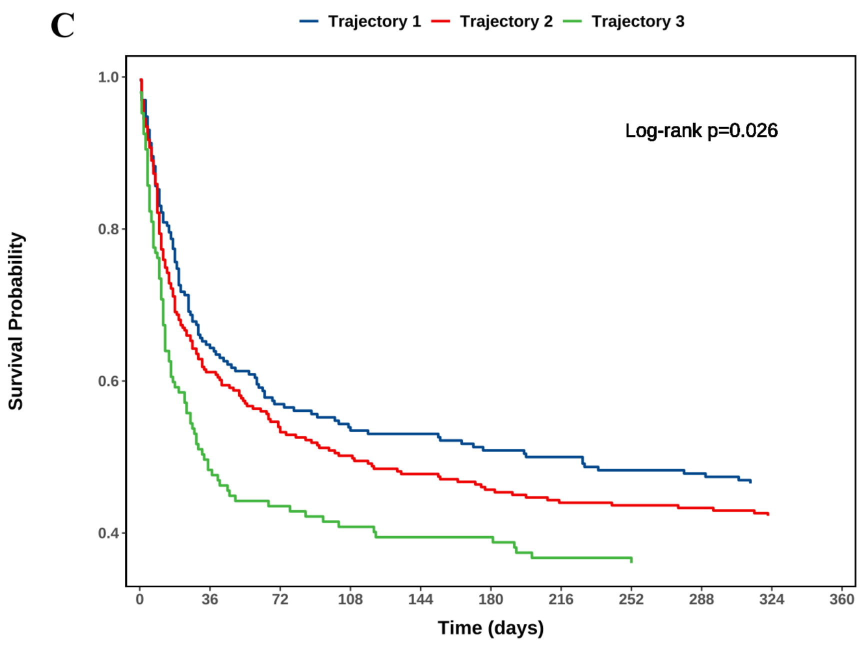

At Risk

Trajectory 1	230	149	131	124	122	117	115	111	110	107	107
Trajectory 2	291	178	157	146	139	133	128	127	126	123	123
Trajectory 3	147	71	64	60	58	58	54	54	53	53	53

Figure S4: Kaplan–Meier curves of 28-day (A), 90-day (B), and 365-day (C) mortality stratified by UCR trajectory groups, in patients with admission creatinine >4 mg/dL and/or RRT.

Table S2 Multivariable Cox regression analysis for different UCR trajectory groups and 28-day, 90-day, and 365-day mortality in patients with admission creatinine >4 mg/dL and/or RRT.

	Crude Model		Model I	
	**HR (95% CI)**	***p***	**HR (95% CI)**	***p***
**28-day mortality**				
Trajectory 1	Reference		Reference	
Trajectory 2	1.15 (0.86–1.56)	0.343	0.99 (0.72–1.35)	0.93
Trajectory 3	1.67 (1.21–2.32)	0.002	1.41 (0.99–2.00)	0.058
**90-day mortality**				
Trajectory 1	Reference		Reference	
Trajectory 2	1.13 (0.88–1.46)	0.34	1.03 (0.79–1.36)	0.805
Trajectory 3	1.55 (1.16–2.07)	0.003	1.41 (1.04–1.93)	0.028
**365-day mortality**				
Trajectory 1	Reference		Reference	
Trajectory 2	1.14 (0.91–1.44)	0.26	1.06 (0.83–1.36)	0.626
Trajectory 3	1.44 (1.10–1.89)	0.007	1.35 (1.01–1.80)	0.042

HR, Hazard Ratio; CI, Confidence Interval.

Crude model: Unadjusted; Model I: Adjusted for gender, age, BMI, congestive heart failure, liver disease, CKD, AKI, sepsis, and albumin infusion.
